# Test for Arsenic

**Published:** 1880-12

**Authors:** 


Test for Arsenic.—The following test is of easy application, and is specially applicable for paper hangings or suspected fabrics: Immerse the suspected paper in strong ammonia, upon a white plate or saucer; then drop a crystal of nitrate of silver into the blue liquid, and if any arsenic be present the crystal will become coated with yellow arseniate of silver, which will disappear on stirring.—*Practitioner.*

